# Ecosystem‐based management of coral reefs under climate change

**DOI:** 10.1002/ece3.4146

**Published:** 2018-05-20

**Authors:** Bethany J. Harvey, Kirsty L. Nash, Julia L. Blanchard, David P. Edwards

**Affiliations:** ^1^ Department of Animal and Plant Sciences University of Sheffield Sheffield UK; ^2^ Centre for Marine Socioecology Hobart TAS Australia; ^3^ Institute for Marine and Antarctic Studies University of Tasmania Hobart TAS Australia

**Keywords:** climate change, coral reef ecology, ecosystem‐based management, marine protected areas, marine spatial planning, resilience, social‐ecological systems

## Abstract

Coral reefs provide food and livelihoods for hundreds of millions of people as well as harbour some of the highest regions of biodiversity in the ocean. However, overexploitation, land‐use change and other local anthropogenic threats to coral reefs have left many degraded. Additionally, coral reefs are faced with the dual emerging threats of ocean warming and acidification due to rising CO_2_ emissions, with dire predictions that they will not survive the century. This review evaluates the impacts of climate change on coral reef organisms, communities and ecosystems, focusing on the interactions between climate change factors and local anthropogenic stressors. It then explores the shortcomings of existing management and the move towards ecosystem‐based management and resilience thinking, before highlighting the need for climate change‐ready marine protected areas (MPAs), reduction in local anthropogenic stressors, novel approaches such as human‐assisted evolution and the importance of sustainable socialecological systems. It concludes that designation of climate change‐ready MPAs, integrated with other management strategies involving stakeholders and participation at multiple scales such as marine spatial planning, will be required to maximise coral reef resilience under climate change. However, efforts to reduce carbon emissions are critical if the long‐term efficacy of local management actions is to be maintained and coral reefs are to survive.

## 1 INTRODUCTION: INCREASING THREATS TO CORAL REEF ECOSYSTEMS

Coral reefs are extremely diverse and valuable ecosystems, providing habitat for a third of marine species in just 0.2% of the ocean (Pandolfi, Connolly, Marshall, & Cohen, 2011). They provide important ecosystem services to over 450 million people living within 100 km of them (Crabbe, 2008; Pandolfi et al., 2011), including fisheries, tourism, building materials, and protection from storm waves and coastal erosion. As such, they are valued at US$350,000 per year for an “average” hectare of coral reef (De Groot et al., 2012).

Yet, coral reefs are under severe threat. Because they exist in coastal waters, they are vulnerable to the effects of human activities, with very few pristine reefs left (Bellwood, Hughes, Folke, & Nyström, 2004; Graham, Cinner, Norstrom, & Nystrom, 2014). An estimated two‐thirds of coral reef fish have been lost compared to historical baselines (Edgar et al., 2014), with fish biomass decreasing closer to major markets (Cinner, Graham, Huchery, & Macneil, 2013; Cinner, Huchery, et al., 2016). Besides local pressures, coral reefs are facing additional threats from anthropogenic carbon emissions.

The amount of carbon dioxide (CO_2_) in the atmosphere has increased from 280 to ~405 ppm since the industrial revolution (Dlugokencky & Tans, 2017), despite a third of all emissions having been absorbed by the oceans (Feely, Doney, & Cooley, 2009). There are two overarching effects of CO_2_ and other greenhouse gas emissions on marine systems. First, upper oceans have already been warmed by 0.11°C per decade between 1971 and 2010 (Rhein et al., 2013), and by 2100 the average temperature is predicted to have increased by 12°C (Scott, 2016). There are likely to be more frequent extreme high‐temperature events, such as those associated with El Niño‐southern oscillation (ENSO) (Frieler et al., 2012). Second, increased emissions have caused lower ocean pH, decreased carbonate ion concentrations and reduced the saturation state of aragonite (Ωa)—together known as “ocean acidification” (OA) (Doney, Fabry, Feely, & Kleypas, 2009; Orr et al., 2005). pH has already declined by 0.1 units (Doney et al., 2009; Orr et al., 2005; Yeakel et al., 2015), with a further reduction of 0.3–0.4 units predicted by 2,100 (Feely et al., 2009; Orr et al., 2005). Furthermore, on a more localised scale, tropical storms are predicted to increase in frequency and intensity due to climate change. Such storms can cause devastating damage to coral reefs, for example accounting for 42% of the coral loss in the Great Barrier Reef (GBR) from 1985 to 2012 (De’ath, Fabricius, Sweatman, & Puotinen, 2012; Emanuel, 2013).

In 2016, record high temperatures caused the third global‐scale mass coral bleaching event—a key consequence of climate change‐affecting 93% of reefs in the GBR, one of the most recognised and well‐managed ecosystems on the planet (ARC Centre of Excellence for Coral Reef Studies, 2016). Corals were hit again by bleaching in 2017, altogether severely affecting two‐thirds of the reef in consecutive years (ARC Centre of Excellence for Coral Reef Studies, 2017) and making this the most extreme bleaching event ever recorded globally. While local managers cannot address the global impacts of ocean warming and acidification, research has sought to provide ways to mitigate against them.

We review recent advances in our understanding of the impacts of ocean warming, acidification and other anthropogenic pressures on coral reef ecosystems, including organism and community responses to their changing environments. We argue the need to integrate ecosystem‐based management and resilience thinking, and then review management and governance strategies for coral reefs under future environmental change. These include the need for climate‐ready marine protected areas (MPAs), alternative mechanisms for reducing local anthropogenic stressors, active management, participatory conservation strategies and marine spatial planning (MSP). We provide an overview of the huge conservation effort necessary to preserve coral reefs and their ecosystem services, and support the communities dependent upon them.

## 2 CLIMATE CHANGE IMPACTS ON CORAL REEF ORGANISMS

Combined, ocean warming and acidification act together to erode the resilience of corals and other reef organisms. These negative impacts thus act from individual to ecosystem levels by reducing survival, recruitment, growth and reproduction, and therefore the potential of corals and reef dwellers to recover from disturbances (Anthony et al., 2011; Ateweberhan et al., 2013).

### 2.1 Climate change impacts on individual performance

Tropical marine organisms generally have narrower ranges of thermal tolerance and exist nearer their upper thermal limits than temperate species (Rummer & Munday, 2017). Temperatures approaching thermal limits have physiological implications for organisms, including increased metabolic rates and reduced aerobic scope, development and growth (Doney et al., 2012; Harvey, Gwynn‐Jones, & Moore, 2013; Ohlberger, 2013). In fish, warming may lead to reduced swimming performance and reproductive capacity (Rummer & Munday, 2017).

The coral–zooxanthellae symbiosis is particularly vulnerable to higher temperatures, which result in the expulsion of the symbiotic algae causing coral bleaching (Carilli, Donner, & Hartmann, 2012; Crabbe, 2008; Hoegh‐Guldberg et al., 2007; Knowlton & Jackson, 2008). Bleaching occurs when temperatures reach 1–2°C above summer maximum temperatures (Hoegh‐Guldberg et al., 2007; Hughes et al., 2003; Jackson, 2010), and can be temporary, leading to decreased coral growth, or prolonged, resulting in mass mortality (Crabbe, 2008; Doney et al., 2012; Veron et al., 2009). Wide‐scale bleaching events have occurred since the 1980s (Baker, Glynn, & Riegl, 2008), but are increasing in frequency and severity, and are expected to become annual events by 2040 (Frieler et al., 2012; Grottoli et al., 2014; Hoegh‐Guldberg et al., 2007; Veron et al., 2009), which would threaten over 90% of reefs (Grottoli et al., 2014).

Ocean acidification impacts corals and reef organisms through different mechanisms than warming. Corals themselves are vulnerable to OA because they require carbonate ions in aragonite form to build their calcium carbonate skeletons; as availability of aragonite decreases so do calcification and growth rates, leading to fragile structures (Doney et al., 2009; Fabry, Seibel, Feely, & Orr, 2008; Pandolfi et al., 2011; Veron et al., 2009). A laboratory study has shown that in addition to reducing calcification, a pH of 7.7 also deformed the skeletal structures of juvenile corals (Foster, Falter, McCulloch, & Clode, 2016). A large decline in calcification by 14.2% in the GBR overall since 1990 has been reported (De’ath, Lough, & Fabricius, 2009), possibly influenced by the mass bleaching events; from 1947 to 2008 inner reefs on the GBR calcification have declined steadily by 0.6% per decade (D’Olivo, McCulloch, & Judd, 2013).

By 2050, reefs are predicted to erode at a faster rate than they can be built, causing net reef dissolution (Silverman, Lazar, Cao, Caldeira, & Erez, 2009). OA reduces coral larvae growth rates leading to higher mortality and lower recruitment (Doropoulos, Ward, Marshell, Diaz‐Pulido, & Mumby, 2012; Munday et al., 2009). The coralline algae on which coral larvae preferentially settle are sensitive to low pH (Doney et al., 2009; Doropoulos, Ward, Diaz‐Pulido, Hoegh‐Guldberg, & Mumby, 2012), and there is evidence that the cues by which coral and fish larvae identify settlement sites are disrupted, suggesting further negative impacts on recruitment (Doropoulos, Ward, Diaz‐Pulido, et al., 2012; Munday et al., 2009).

It is possible that increased temperatures may mitigate somewhat against the reduced growth and calcification caused by acidification (Foster, Gilmour, Chua, Falter, & McCulloch, 2015; Foster et al., 2016). However, overall the combined effects of warming and acidification are likely to be damaging (Hoegh‐Guldberg, Poloczanska, Skirving, & Dove, 2017). Calcification and growth rates are decreased further by extreme temperatures, coral bleaching and disease; reduced calcification in turn makes corals more susceptible to bleaching and disease (Anthony, Kline, Diaz‐Pulido, Dove, & Hoegh‐Guldberg, 2008; Chauvin, Denis, & Cuet, 2011; De’ath et al., 2009; Grottoli et al., 2014; Rodrigues & Grottoli, 2006; Veron et al., 2009). One study found that high CO_2_ dosing (~pH 7.65) led to a two‐ to threefold increase in bleaching, a reduction in productivity to near zero, and 130%–190% decrease in calcification relative to controls, in three coral species (Anthony et al., 2008). Coral diseases are exacerbated by bleaching, elevated temperatures and acidification; they also make corals more susceptible to bleaching, and can delay recovery from it (Baker et al., 2008; Bruno et al., 2007; Harvell, 2002).

Skeletal damage from reduced calcification and growth can make corals more sensitive to the structural damage caused by bioeroders and storms (Anthony, 2016; Baker et al., 2008; Veron et al., 2009), which models predict will also increase in intensity by 45% and frequency by 10%–40% under climate change (Emanuel, 2013). In the Caribbean, 177 sites experienced an average 17% reduction in coral cover in the year after a hurricane (Gardner, Côté, Gill, Grant, & Watkinson, 2005), although storms could mitigate against bleaching by mixing and cooling waters on a large scale (Carrigan & Puotinen, 2014).

The combined effects of ocean acidification and warming differentially affect species and their life stages, with early life stages generally more susceptible. For example, a meta‐analysis has shown larvae of multiple taxa have higher warming‐induced mortality than adults (Harvey et al., 2013). Understanding how individual‐level responses to warming and OA influence population, community and ecosystem structure and dynamics is a key research area in both terrestrial (Nadeau, Urban, & Bridle, 2017) and marine ecology (Ohlberger, 2013).

### 2.2 Reef responses to climate change

**Figure 1:**
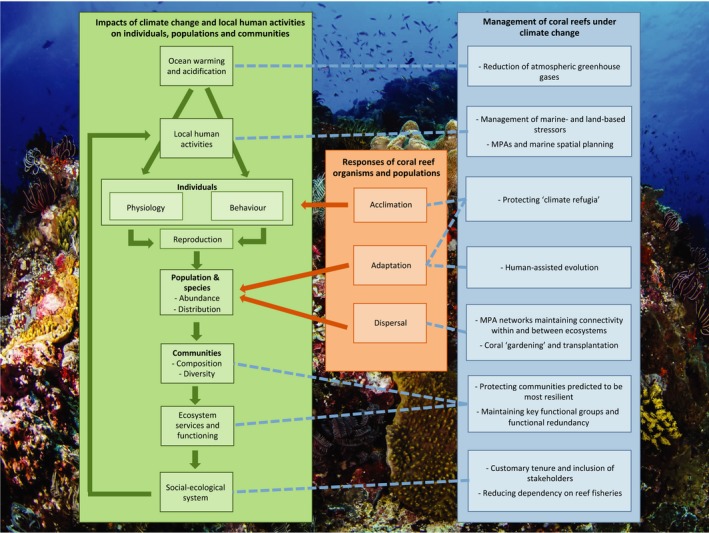
Flow diagram of the impacts of climate change and human activities on coral reefs from individuals to populations, communities, ecosystem functioning and socialecological systems (green), and coral reef organisms’ responses to mitigate the effects of climate change (orange). The direct impacts of climate change and local human activities on individuals can be multiplied through changes to physiology, behaviour and, therefore, reproduction, and this, combined with species‐specific responses to climate change (acclimation, adaptation and dispersal) influence populations, communities and ultimately ecosystem functioning. The response of socialecological systems to climate change may also then influence local anthropogenic pressures in unanticipated ways. Management strategies aimed at reducing the impacts of climate change and local anthropogenic pressures, facilitating positive responses to them, and maintaining the resilience of communities and socialecological systems are shown in blue, with dotted blue lines linking management strategies to their level of direct impact on threats, and from organism to socio‐ecological scales. Photograph of Gili Mimpang coral reef, Bali, Stefan Follows (http://www.kpnphotographic.com/)

Coral reef organisms may adapt, acclimate or disperse to mitigate the impacts of climate change (Hoegh‐Guldberg, 2014; see Figure 1). Coral reefs are found under variable environmental conditions (Carilli et al., 2012; Freeman, Kleypas, & Miller, 2013) and can thrive in some areas with high temperatures and acidity (Fabricius et al., 2011; Oliver & Palumbi, 2011; Shamberger et al., 2014), suggesting adaptive potential. Furthermore, corals previously exposed to higher or more variable temperatures are more resistant to bleaching in both field and laboratory studies (Carilli et al., 2012; Crabbe, 2008; Middlebrook, Hoegh‐Guldberg, & Leggat, 2008; Silverstein, Cunning, & Baker, 2015). Transgenerational acclimation, whereby offspring of parents exposed to altered environmental conditions are better suited to those conditions, has been observed in both fish and coral species, although the mechanisms by which this happens and the degree to which it could enable coral reef species to survive future environmental conditions, remain uncertain (Rummer & Munday, 2017; Torda et al., 2017).

One method of acclimation is a change in the coral–algae symbiosis to more heat‐resistant algae (Parmesan, 2006). Experimentally bleached corals recovered with a higher proportion (>90%) of stress‐tolerant symbiotic algae, reducing damage in subsequent bleaching events (Silverstein et al., 2015). It is unknown however whether this is enough to support coral survival under highly stressful conditions, or whether the unprecedented rate of environmental change is too rapid to enable successful adaptation or acclimation (Hoegh‐Guldberg, 2014; van Oppen, Oliver, Putnam, & Gates, 2015). A recent review by Hoegh‐Guldberg et al. (2017) suggests that there is little evidence to show corals can adapt fast enough, especially considering their long generation times.

An alternative to adaptive responses to environmental change is dispersal to maintain suitable conditions (Doney et al., 2012; Freeman et al., 2013; Jackson, 2010; Simpson, Blanchard, & Genner, 2013). Large numbers of tropical marine species are predicted to move to higher latitudes, (Cheung et al., 2009) and some coral reef species’ ranges have already shifted (Baird, Sommer, & Madin, 2012; Graham et al., 2014; Precht & Aronson, 2004), including four coral species rapidly expanding their northern ranges by 14 km per year in Japan (Yamano, Sugihara, & Nomura, 2011). However, generally the range of suitable coral reef habitat will shrink due to limiting factors such as temperature, light availability and aragonite saturation state (Freeman et al., 2013). Indeed, temperature and acidification have opposite latitudinal gradients, so coral reef ecosystems cannot shift to escape the effects of both (Van Hooidonk, Maynard, Manzello, & Planes, 2014). In tropical and subtropical coral reefs in Japan, suitable coral habitat will disappear by the 2040s under future projected temperature and acidification changes (Yara et al., 2012). Overall, while some individual species may disperse, it is unlikely that whole coral reef ecosystems will be able to shift to escape the effects of climate change (Hoegh‐Guldberg et al., 2017). Understanding and predicting climate‐change driven range shifts is a key area of research in both terrestrial and marine ecosystems (Bonebrake et al., 2017).

Climate change also impacts on coral reef ecosystems through the interactions of individuals, populations and communities (see Figure 1). Corals are ecosystem engineers upon which the entire reef community depend: they build the ecosystem framework, providing habitat for other organisms (Wild et al., 2011). The loss of habitat structural complexity due to coral reef dissolution and frequent bleaching can reduce the prevalence of other groups of organisms (Fabricius, De’ath, Noonan, & Uthicke, 2014), reduce fish biomass and abundance by threefold (Rogers, Blanchard, & Mumby, 2014) and change reef functioning (Wild et al., 2011).

Organism responses to climate change such as reduced reproductive capacity in fish due to warming can have population‐level consequences (Rummer & Munday, 2017), while species‐specific responses to climate change such as differential adaptation and dispersal potential result in altered biotic interactions, trophic cascades, loss of key species and disruption of metapopulation dynamics, impacting on populations and communities (Doney et al., 2012; Graham et al., 2014; see Figure 1). For example, a Caribbean coral (*Porites astreoides*) unable to acclimate had 14%–61% lower energy reserve concentrations than corals which did acclimate after a repeat bleaching event: corals unable to acclimate are more susceptible to local extinction in the future, reducing overall reef biodiversity (Grottoli et al., 2014). Climate change also influences biotic interactions with negative consequences for coral: OA accelerated bioerosion of coral by a sponge (*Cliona orientalis*) by 61% under lower pH conditions in one study (Wisshak, Schonberg, Form, & Friewald, 2012), while increasing temperatures by 2°C promoted 4.2–4.9 times higher recruitment (and therefore coral predation) by coral crown‐of‐thorns starfish (*Acanthaster planci* or CoTS), a major issue in the GBR (Uthicke et al., 2015).

These climate change‐associated changes in community composition and biodiversity of coral reefs can ultimately lead to regime shifts or “novel ecosystems”, either non‐coral ecosystems or alternative coral communities dominated by thermally resistant species (Graham et al., 2014). While exactly predicting regime shifts is challenging, there are several conditions known to reduce coral reef resilience including herbivore removal, low reef structural complexity and high nutrient concentrations (Fabricius, 2005; Graham, Jennings, MacNeil, Mouillot, & Wilson, 2015; Graham, Nash, & Kool, 2011; Hughes, Graham, Jackson, Mumby, & Steneck, 2010; Hughes et al., 2007; Mumby, Hastings, & Edwards, 2007). Through differential mortality, range shifts and invasive species, ecosystems are changing and the functional consequences of these changes are largely unknown, leaving the future of coral reefs uncertain (Graham et al., 2014).

## 3 INTERACTIONS BETWEEN CLIMATE CHANGE FACTORS AND LOCAL HUMAN ACTIVITIES

While climate change alone is a major threat to coral reefs, it cannot be considered in isolation when local human activities are severely damaging coral reef ecosystems (Burke, McManus, Spalding, & Perry, 2011; Halpern et al., 2008; see Figure 1). These include direct damage and sedimentation from coastal development, pollution from land‐use change, increased nutrients from agriculture, invasive species, overfishing and destructive fishing practices (Burke et al., 2011). These factors exacerbate the negative impacts of climate change on coral reefs through direct destruction of reefs and their structural complexity, increased bleaching susceptibility of corals, reduced coral recruitment and growth, and increased disease prevalence (Ateweberhan et al., 2013; Bijma, Portner, Yesson, & Rogers, 2013; Halpern et al., 2008). Table 1 provides some key examples of these harmful interactions for four major threats of sedimentation, nutrient enrichment, overfishing and destructive fishing. Due to these additional pressures, reefs are becoming less resistant to chronic climate change stressors, and are less able to recover from related disturbances such as bleaching, storms and CoTS outbreaks (Anthony et al., 2011; Hughes et al., 2007; McClanahan, Graham, & Darling, 2014).

**Table 1:** Key examples of local stressors and their interactions with climate change factors

Local stressor	Interaction with climate change factors	References
Destructive fishing practices such as blast (dynamite) and cyanide fishing, and destructive types of fishing gear, e.g., gill nets	Destruction of reef structure and reduction in structural complexity, which is also driven by climate change	Fox and Caldwell (2006), Graham et al. (2006) and Burke et al. (2011)
	Coral bleaching and mortality exacerbated by cyanide	Jones and Hoegh‐Guldberg (1999) and Burke et al. (2011)
Overfishing. Globally, coral reef fisheries landings are 64% higher than is sustainable (Newton et al., 2007)	Removal of herbivores increases algal growth, reducing space for coral growth and recruitment, and thus coral recovery after bleaching events	Hughes et al. (2007), Jackson (2010), Doney et al. (2012), Ateweberhan et al. (2013) and Wiedenmann et al. (2013)
	Macroalgal growth also increases prevalence of coral diseases and experimentally induces 100% coral mortality through increasing microbes in the water	Smith et al. (2006) and Sandin et al. (2008)
Sedimentation	Causes higher turbidity in coastal waters and leads to a reduction in light availability to corals (e.g., a 20% reduction in mean annual light availability in the GBR in wetter years), and therefore photosynthesis and growth	Fabricius (2011) and Fabricius, Logan, Weeks, and Brodie (2014)
	Sedimentation is associated with higher abundance of macroalgae, by up to five times in one study in the GBR	De’ath and Fabricius (2010)
	Reefs exposed to higher durations of sediment plumes had double the amount of diseases compared to nearby reefs with little or no exposure	Pollock et al. (2014)
	Sedimentation reduces coral recruitment through reduced larval survival and lower abundance of coralline algae (60% in one laboratory study (Harrington, Fabricius, Eaglesham, & Negri, 2005))	Fabricius and De’ath (2001), Harrington et al. (2005) and Perez, Rodgers, Jokiel, Lager, and Lager (2014)
Nutrient enrichment	Higher concentrations of dissolved inorganic nutrients do not directly kill corals but can reduce coral calcification and growth	Fabricius (2005, 2011), Chauvin et al. (2011), Ateweberhan et al. (2013) and Wiedenmann et al. (2013)
	Coral diseases are also more (2–5× experimentally) prevalent under nutrient enrichment, which can exacerbate existing infections	Bruno, Petes, Harvell, and Hettinger (2003), Voss and Richardson (2006), Pollock et al. (2014) and Vega Thurber et al. (2014)
	Nutrient enrichment leads to higher phytoplankton concentrations and therefore turbidity, decreasing zooxanthellae photosynthesis	Fabricius, 2011
	Increases nutrient‐limited competitive macroalgae	Fabricius (2011) and D’Angelo and Wiedenmann (2014)
	Leads to higher frequency of CoTS outbreaks, because CoTS larvae are nutrient‐limited. Modelled CoTS outbreaks in the GBR have increased from one in 50–80 years to one every 15 years due to increased nutrient loading (Fabricius, Okaji, & De’ath, 2010)	Brodie, Fabricius, De’ath, and Okaji (2005) and Fabricius et al. (2010)
	Higher nitrogen levels stimulate growth of zooxanthellae; however when phosphorus‐limited, and combined with heat and light stress, this can increase bleaching. Vega Thurber et al. (2014) found a 3.5‐fold increase in bleaching frequency in corals exposed to higher levels of nitrogen and phosphorous experimentally. Nutrients therefore increase coral susceptibility to bleaching and lower “bleaching thresholds”	Veron et al. (2009), Wooldridge (2009), Wagner et al. (2010), Ateweberhan et al. (2013), Wiedenmann et al. (2013) and Vega Thurber et al. (2014)

These interacting factors may be antagonistic (the combined impact is less than the sum of their individual impacts), additive (the combined impact is equal to the sum of individual impacts) or synergistic (the combined impact exceeds the sum of individual impacts) (Ban, Graham, & Connolly, 2014). Ban et al. (2014) synthesised all available studies on interactions between two or more stressors on coral reefs, finding that sedimentation, storms and temperature influenced the highest number of other stressors, while the most‐influenced stressors were nutrients, CoTS and pathogens. Most stressor‐stressor interactions were either additive or synergistic.

However, there are still very little data on most interactions, especially those where variables are not easy to control, such as fishing effort as this is often unmonitored (Ban et al., 2014). Disentangling the interacting impacts of a variety of stressors on such a complicated ecosystem is highly challenging and may not be possible (Côté, Darling, & Brown, 2016). More research is needed to understand interactions between stressors, especially those which most influence other stressors, to enable more effective management prioritisation to mitigate against the most severe impacts of climate change and human activities on coral reef ecosystems (Ban et al., 2014).

## 4 CORAL REEF MANAGEMENT IN A CHANGING ENVIRONMENT

### 4.1 Towards ecosystem‐based management of coral reefs

Conservation goals for coral reefs have traditionally focused on reversing declines and returning the ecosystem to “pristine” conditions (Rogers et al., 2015). However, many people rely on reefs for food and livelihoods and multi‐use of reefs requires careful consideration of the identification and understanding of trade‐offs among conflicting management and conservation objectives. This also requires a detailed understanding of the multiple stakeholders involved in direct use of and those involved in distal activities that could be impacting reefs, for example, in the case of land‐based pollution (Fredston‐Hermann et al., 2016) and logging (Hamilton et al., 2017). The type of management and conservation measures in place needs to take into account a combination of the changing environmental and human impacts as well as their potentially changing benefits.

Marine protected areas or no‐take marine reserves are the most common form of reef management, focusing predominantly on reducing local anthropogenic pressures (Agardy, di Sciara, & Christie, 2011; Mellin, Aaron MacNeil, Cheal, Emslie, & Julian Caley, 2016). The recent trend in designations of very large, remote MPAs such as the 640,000 km^2^ Chagos MPA (Jones & De Santo, 2016) to meet the 10% coverage target of the Convention on Biological Diversity may represent political rather than science‐based interests. These MPAs cost little to designate but may not be effective or representative (Jones & De Santo, 2016). They do not protect the most threatened reefs—those associated with high human population densities—nor address social issues such as maintaining ecosystem services for those reliant on reefs for food or income (Sale et al., 2014). Their designation should not divert attention from management of more damaged coral reefs in highly populated areas.

There is increasing recognition that coral reef management has been unable to halt degradation, and that returning to “pristine” conditions is an unrealistic goal (Abelson et al., 2016; Burke et al., 2011; Hughes et al., 2017; Rinkevich, 2015), particularly in the context of changing environmental conditions. Conservation is moving away from this more traditional approach, as research seeks to provide conservation practitioners with a more diverse toolbox, including a range of indicators (Nash & Graham, 2016) and modelling frameworks (McClanahan et al., 2012). The focus of management has changed from individual species and selected reefs, to management of the whole seascape, as the complexity of reef ecosystem responses to climate change and local human stressors has been realised (McClanahan, Graham, Macneil, & Cinner, 2015; Olsson, Folke, & Hughes, 2008; Weijerman et al., 2015). Ecosystem‐based management (EBM) is a more holistic framework, incorporating interactions within ecosystems and socialecological links, impacts of multiple stressors on key functional groups and ecosystem processes, with the goal of maintaining ecosystem functioning and services (Levin & Lubchenco, 2008).

The concept of resilience has been advocated and can be used to support the design and implementation of EBM (Hughes et al., 2017; Levin & Lubchenco, 2008). Ecological resilience can be defined as the ability of an ecosystem to maintain the same structure and functioning in a changing environment, including resistance to stress and recovery from disturbances (Levin & Lubchenco, 2008; McClanahan et al., 2012). One of the benefits of embedding this framework into EBM for reefs is that resilience of coral reefs has been relatively well studied, for example, in the context of recovery following disturbance events such as bleaching (Anthony, 2016) and socio‐ecological resilience (Cinner, Pratchett, et al., 2016; Kittinger, Finkbeiner, Glazier, & Crowder, 2012). Vulnerability frameworks that integrate across ecological, social and economic aspects of coral reefs are an important part of developing adaptive and more integrated ecosystem management strategies under climate change (Cinner, Huchery, et al., 2013).

In the context of climate change, changes in underlying reef resilience could also occur if key recovery processes (such as recruitment, growth or dispersal) are impacted by climate change (Anthony, 2016). Management strategies could therefore enhance resilience to ongoing stressors (e.g., warming, sedimentation) and act directly to reduce the impacts of disturbances, such as control of CoTS (Anthony et al., 2015). Reducing local drivers of coral reef decline may increase the chances of re‐establishment of coral‐dominated reefs from degraded reefs which are themselves resistant to change (Graham et al., 2013). Maintaining resilience also means retaining diversity and functional redundancy in the face of change, managing ecosystem connectivity, understanding and managing feedbacks, and promoting social principles including encouraging education, broadening participation and advocating integration of multiple stakeholders (Biggs et al., 2012; Hughes et al., 2017).

To maintain the resilience of social‐ecological systems to climate change, we advocate for the need to (1) minimise local stressors, (2) design MPAs that address not only local pressures but also incorporate measures to address global environmental change, (3) utilise active management approaches such as human‐assisted evolution and reef restoration, and (4) develop coordinated management and governance at multiple scales from local customary tenure and community participation, to broader‐scale MSP, to regulations at the international scale. Each of these approaches is considered in depth below.

### 4.2 Minimising local stressors

Reducing local anthropogenic impacts may make coral reefs more resilient to climate change and OA by reducing the harmful interaction with climate change factors (see Table 1) (Hoegh‐guldberg & Bruno, 2010; Hughes et al., 2003; IGBP, 2013; Knowlton & Jackson, 2008; Pandolfi et al., 2011). Reducing local stressors also buys time for coral reef species to adapt or acclimate (Hoegh‐Guldberg, 2014), especially in more thermally resistant coral reefs with higher adaptive potential (e.g., Carilli et al., 2012), such as in the northern Red Sea and Arabian Gulf (Coles & Riegl, 2013; Fine, Gildor, & Genin, 2013).

A long‐term (20 years), large‐scale (150,000 km^2^) study of coral reef communities on the Great Barrier Reef provided strong evidence that reducing local anthropogenic impacts might make coral reefs more resilient to a range of stressors, including coral bleaching (Mellin et al., 2016). This was seen through 21%–38% higher stability of reef community composition, lower susceptibility to initial impacts, and 20% increased recovery times (Mellin et al., 2016). A simulation model for Bolinao in the Phillipines found that management of water quality, and to a lesser extent fishing, can significantly improve reef state under future climate change scenarios, by enhancing recovery after bleaching events (Gurney, Melbourne‐Thomas, Geronimo, Alino, & Johnson, 2013).

Increasing water quality through minimising sedimentation and nutrient enrichment could improve ecosystem health within decades (Fabricius, De’ath, McCook, Turak, & Williams, 2005). Better watershed management through improved agricultural practices near rivers, restoration of riparian reserves along rivers and of coastal floodplains and returning to more natural flow regimes could reduce run‐off and nutrient enrichment (Kroon, Schaffelke, & Bartley, 2014). Regulation of waste water could reduce bleaching severity (Wagner, Kramer, & Van Woesik, 2010), while minimising sedimentation should reduce coral diseases (Pollock et al., 2014) and decreasing nutrient loads could allow more time for the adaptation of the coral–zooxanthellae symbiosis to global change (Wooldridge, 2009).

On eliminating fishing in protected areas, reef resilience may be increased through a number of mechanisms: herbivory, reduced coral predation, maintaining structural complexity and increased response diversity, and promoting recovery from bleaching events (Adam, Burkepile, Ruttenberg, & Paddack, 2015; Graham & Nash, 2013; Graham et al., 2015; Mumby et al., 2007; Nash, Graham, Jennings, Wilson, & Bellwood, 2015). No‐take marine reserves control fishing effort and enable fish populations to recover (Burke et al., 2011), but enforcement is often limited and reserves do not fulfil their function (McClanahan, Marnane, Cinner, & Kiene, 2006; McClanahan et al., 2015). Determining the carrying capacity of fish biomass in relation to environmental and anthropogenic variables could help managers to develop reference levels that would reduce trade‐offs between fisheries’ objectives and the protection of reef function (Valdivia, Cox, & Bruno, 2017).

Alternative fishing restrictions, such as controlling species caught, fishing gear and access, and seasonal closures of breeding sites, can be successful at sustaining fish biomass while maintaining key ecosystem functions such as herbivory (Burke et al., 2011; MacNeil et al., 2015; McClanahan et al., 2015; Nash, Abesamis, Graham, McClure, & Moland, 2016). Setting fisheries targets is challenging due to the multiple species with diverse ecological functions, however total catch biomass alone can provide easy, effective targets where more information is not available (McClanahan et al., 2015). Climate change and certain fishing gears may simultaneously select for functionally important species. For example, spear guns target specific herbivorous species that are susceptible to climate change, and as a result, restrictions on spear‐guns could be a targeted strategy for supporting reefs under pressure from climate change (Cinner et al., 2009). A key challenge is the quantification of the full suite of ecological, social and economic trade‐offs associated with changes in any of the management levers that are used, but this could be addressed using management strategy evaluation (Fulton, Smith, Smith, & Johnson, 2014).

### 4.3 Marine protected areas

The efficacy of current MPAs is debatable. A meta‐analysis of 310 MPAs found coral cover remained stable within MPAs but declined in fished areas outside, while marine reserves can successfully maintain reef fish biomass, but only in high compliance with MPA regulations (Cinner, Huchery, et al., 2016). A recent study in the GBR suggests MPAs can increase coral reef resilience to natural disturbances exacerbated by climate change, such as coral bleaching, disease and storms (Mellin et al., 2016), while in the Seychelles recovery after a bleaching event was faster within MPAs provided macroalgal cover was low (Wilson et al., 2012). However, several studies have not shown measurable positive impacts (Bruno & Valdivia, 2016; Graham et al., 2008, 2015; Selig, Casey, & Bruno, 2012), and many MPAs are small, with low enforcement and compliance, and as such do not achieve management objectives (Norström et al., 2016; Sale et al., 2014).

Given limited conservation resources (McCarthy et al., 2012) and the time needed to produce positive benefits within MPAs (McClanahan & Graham, 2015), investing in MPAs without considering future threats may result in them becoming less effective over time (McLeod, Salm, Green, & Almany, 2009). If MPAs are to support the resilience of coral reefs in the face of climate change, providing time for acclimation, adaptation or dispersal by reef species, forward looking design is critical (Ban et al., 2011; Lawler, Watson, & Game, 2015; McLeod et al., 2009). Although research has highlighted general characteristics of effective MPAs today (no‐take, enforced, old, large and isolated) (Edgar et al., 2014), other factors need to be considered in a high‐CO_2_ world. MPA design needs to address future scenarios in addition to present issues (Makino et al., 2014, 2015).

New MPAs should protect key areas under a range of future environmental scenarios. Key areas include locations where change is more gradual (“climate refugia”), ecologically important areas including fish spawning aggregations, and areas predicted to be more resilient in the long‐term, such as those with high herbivore populations and low sedimentation (Ban et al., 2011; Graham et al., 2008; Groves et al., 2012; McLeod et al., 2009). Recent research has also highlighted the importance of protecting degraded reefs in addition to healthy, resilient ones, due to their increasing percentage of global reef area (Abelson et al., 2016).

Small, unconnected MPAs are unsustainable as the surrounding seascape also needs to be managed, often in MPA networks (McLeod et al., 2009). MPA networks in particular should replicate habitat types within them (“spreading risk”), maintain ecological connectivity to facilitate recruitment and dispersal, and sustain not just biodiversity but ecosystem functioning and services, and key functional groups such as herbivorous fish (Groves et al., 2012; Lawler et al., 2015; McLeod et al., 2009; Mumby & Steneck, 2008). MPA placement should therefore reflect both current and future species distributions where ranges are expected to change, to facilitate dispersal (Makino et al., 2014).

Implementing MPAs that have been effectively designed to meet climate change objectives is not always sufficient because implementation does not always equate to compliance (McClanahan et al., 2006). A total of 70% of coral reefs are found in developing nations with low enforcement capacity (Ban et al., 2011); thus, MPAs may suffer from poor compliance (McClanahan et al., 2006), which can have serious consequences for their effectiveness (McClanahan & Graham, 2015). Including local stakeholders in MPA planning and design may drive improved levels of compliance and improve reef resilience outcomes (Cinner, Huchery, et al., 2016). Bottom‐up, participatory management and conservation approaches that account for the local context are central to successful management outcomes on many coral reefs, particularly in developing nations (Christie et al., 2009; Norström et al., 2016).

MPAs that are planned, designed and implemented using participatory approaches and with explicit climate change objectives represent an important strategy in marine management. The benefits of an MPA could spill over into surrounding areas supporting fisheries (Harrison et al., 2012; Hughes et al., 2010; McCook et al., 2010), although benefits are unlikely to be distributed evenly, introducing issues of inequity (Cinner et al., 2014; Daw et al., 2015). Furthermore, MPAs are unlikely to be a cure‐all for coral reef conservation because they do not provide direct protection from external impacts such as sedimentation from land run‐off (Fabricius et al., 2005; Gilby, Maxwell, Tibbetts, & Stevens, 2015), and may only be effective at maintaining ecosystem services in reefs of high structural complexity (Rogers et al., 2015). Where structural complexity is lost, reefs are heavily degraded, climate change impacts are unpredictable and there is high reliance on reefs, MPAs will need to be combined with other management approaches (Abelson et al., 2016; Graham et al., 2013; Makino et al., 2015; Rogers et al., 2015).

### 4.4 Active management approaches

On degraded reefs, removal of human pressures may be insufficient to facilitate recovery to coral‐dominated ecosystems, suggesting that active reef restoration may be necessary to maintain healthy ecosystems more resilient to climate change (Abelson et al., 2016; Adam et al., 2015; Rogers et al., 2015). Introducing artificial complexity to replace the lost habitat structures of low complexity reefs, for example due to acidification, has been successful at small scales such as in the Gulf of Aqaba, Red Sea (Al‐Horani & Khalaf, 2013; Rogers et al., 2015). Coral “gardening”, where corals are farmed in mid‐water structures then transplanted to colonies, has been successful, leading to increased reproduction and larval dispersal (Rinkevich, 2014), for example, a 35% increase in oocyte production in farmed coral in Eilat, Red Sea (Amar & Rinkevich, 2007). Furthermore, coral transplantation could be used to reduce tourism exposure to natural reefs (Rogers et al., 2015). These techniques are unlikely to provide larger‐scale solutions, yet can be effective locally.

Human‐assisted evolution of coral reef organisms is another novel management tool, whereby genetically modified stress‐tolerant corals are transplanted onto reefs (Anthony, 2016; van Oppen et al., 2015). This could also increase organisms’ resilience to environmental change by increasing resistance to stressors such as warming and acidification, and recovery from disturbances (van Oppen et al., 2015), and could maintain important ecosystem services provided by corals (Anthony, 2016). However, it could lead to a monopoly of a few resistant species, reducing overall biodiversity (Anthony, 2016).

### 4.5 Combining different tiers of conservation management with spatial planning

Conservation management cannot focus solely on biodiversity measures or maintaining pristine reefs in remote locations. Six million fishers from nearly 100 countries rely on coral reefs for their livelihoods (Teh, Teh, & Sumaila, 2013); therefore, management needs to support the resilience of both coral reefs and the communities that depend on them (Anthony et al., 2015; Cinner, Pratchett, et al., 2016). Climate change impacts on reef social‐ecological systems are bi‐directional: how climate change impacts on communities determines how they then interact with the environment. For example, if climate change and local human pressures reduce local fish populations, will fishers look further afield for more fish, or turn to alternative incomes (Cinner, Pratchett, et al., 2016)? Such impacts are poorly understood, so the resilience of social‐ecological systems to climate change cannot be predicted.

Reducing dependency on fishing is imperative to protecting coral reefs, but highly challenging in societies with population growth, a lack of alternative income sources and poor governance (Burke et al., 2011; Newton, Côté, Pilling, Jennings, & Dulvy, 2007). These factors cannot be managed by conservation practitioners; however, management strategies built upon access rights, particularly, customary tenure are gaining traction (Christie et al., 2009; Jupiter, Cohen, Weeks, Tawake, & Govan, 2014). A key study of over 2,500 reefs globally showed that reefs doing better than predicted from their environmental and social context commonly had customary management schemes and marine tenure (Cinner, Huchery, et al., 2016). In contrast, reefs doing worse than predicted had technological commonalities such as intensive netting methods (Cinner, Huchery, et al., 2016). These findings suggest reef management may benefit from integrating customary and/or marine tenure arrangements, and gear restrictions rather than focusing solely on MPAs. These arrangements arise within indigenous communities or kinship groups and restrict certain behaviours, for example fishing at specific times or locations. Importantly, these restrictions may support key reef functions and processes, with positive implications for coral reef resilience (Aswani, Albert, Sabetian, & Furusawa, 2007).

Further considerations often overlooked are large‐scale or “distal” human drivers of coral reef degradation, which influence local anthropogenic threats such as fishing and pollution (Hughes et al., 2017). These include international demand for certain species for food or the aquarium trade; increasing human migration (which may be exacerbated by climate change); foreign investments driving land use change; and changing fishing effort (Cinner, Graham, et al., 2013; Norström et al., 2016; Rhyne et al., 2012; Sale et al., 2014). Broader management strategies, especially marine spatial planning, need to be used to combat these external drivers. MSP incorporates MPAs and zoning, supports key ecosystem services, and separates conflicting uses of marine resources, for example by dividing coastal waters for different activities (e.g., conservation, food security and livelihoods) and integrating local and regional targets (Agardy et al., 2011; Sale et al., 2014). This approach compensates for many of the deficits of MPAs on their own, incorporating them into a broader system of management and preventing degradation of surrounding areas (Agardy et al., 2011). It should also reduce conflicts, maintain ecosystem services, and can facilitate rights‐based governance (Sale et al., 2014). For instance, models for a national spatial plan for Belize which quantified ecosystem services led to a 25% increase in coastal protection with increases in tourism and doubling of fishing revenue when contrasted with models only including stakeholder views (Arkema et al., 2015).

Prioritisation of goals at national and regional scales, the inclusion of ecosystem services, and broad, coordinated conservation planning are essential to protecting coral reefs. Successful implementation of MSP will require adaptation to local need and governance, participation of communities and stakeholders, political motivation at national and international levels, and support from NGOs and international development partners. Internationally, regulation of markets and the aquarium trade could reduce demands on coral reefs by ensuring fish are caught through sustainable measures (Burke et al., 2011). For example, international screening for the effects of cyanide poisoning on live fish could promote sustainable fishing (Calado et al., 2014). From local, rights‐based management, through to regional and national marine planning and international laws and regulations on trade, coral reef conservation is a multi‐scale objective requiring support from a wide variety of stakeholders and organisations.

## 5 CONCLUSIONS

Coral reefs are severely threatened by a suite of human‐induced stressors at local and global scales. Ecosystem‐based management combined with resilience thinking can be used to better effect than approaches which do not take into account the multi‐use, complex social‐ecological nature of coral reef systems. Improving MPA design to enable coral reef organisms to adapt, acclimate or disperse under climate change is necessary but not sufficient: a range of other conservation tools will need to be employed including management of external stressors, alternative fisheries restrictions, novel approaches such as active restoration, inclusion of social–ecological factors and action on multiple scales. Figure 1 summarises various management strategies aimed at reducing the impacts of climate change and local human activities, facilitating positive responses to them via acclimation, adaptation or dispersal, and sustaining ecosystem services and functioning to maintain the resilience of social‐ecological systems.

To save coral reefs in the long‐term, global action to reduce carbon emissions and limit warming to 1.5‐2°C is vital (Frieler et al., 2012; Hoegh‐guldberg & Bruno, 2010; Hoegh‐Guldberg et al., 2017). However, integration of MPAs with other management strategies and participatory approaches provide the best chance of maximising coral reef resilience in the face of climate change, while ensuring equitable access to the valuable ecosystem services they provide.

## CONFLICT OF INTEREST

None declared.

## AUTHOR CONTRIBUTION

All listed authors meet the four criteria in the ICMJE guidelines on authorship. Bethany Harvey drafted the first version, while all authors contributed ideas to the concepts and edited various drafts. Kirsty Nash specifically wrote a section on ecosystem‐based management, Julia Blanchard contributed various paragraphs, while David Edwards edited many times. All authors approved of the final version to be published, and all agree to be accountable for all aspect of work contained within.
